# P06-13 Movida.Cronos – eHealth app at primary care to fight sedentary behavior

**DOI:** 10.1093/eurpub/ckac095.098

**Published:** 2022-08-29

**Authors:** Pedro Morouço, Bruno Carreira, Rui Pinto

**Affiliations:** Polytechnic Institute of Leiria, Leiria, Portugal

**Keywords:** Mobile application, Sedentary behavior, Physical Exercise Adherence

## Abstract

**Background:**

Health systems play a fundamental and recognized role in promoting physical activity, particularly in the context of primary health care. eHealth is a broad concept that incorporates any area that combines healthcare and technology. This concept is often associated with improvements in process efficiency and cost reduction. Still, high occurrence of physical inactivity demonstrate the importance of developing new eHealth approaches for a more effective actuation. The aim of this work was to develop a new methodology to increase physical activity level, at primary health care.

**Methods:**

A mobile application with web platform was developed to: physical activity prescription and monitoring; registration of biomedical variables, sending messages to users, managing physical activity events at the initiative of users (social network). The application was explained to the medical doctors and data from a case-study was accessed (56 years-old male, Caucasian, single, nuclear family, upper middle class). History of physical inactivity, obesity, hypertension, dyslipidemia and depressive disorder (sertraline 50mg and mexazolam 1mg therapy), body composition, physical and CRP fitness were assessed. International physical activity and Self-perceived quality of life assessment questionnaires were also used.

**Results:**

It was possible to understand that just by talking was not enough to engage the patient on a more active lifestyle. After several failed attempts on counseling for lifestyle changing, patient was referred for using the developed app. Physical exercise tailored plan was prescribed using the Movida.cronos. This tool opened the possibility of assessing patient performance and adherence to the prescribed program (>90%). It allowed a narrow informative channel between the patient and the medical doctor. In three months, the patient reduced form obesity to overweight (-3.4%), decrease his waist circumference (-6.3%), fat mass (-1.8%) and arterial mean pressure (-5.1%); with an associated increase on step test performance.

**Conclusions:**

This case study demonstrate the potential use of eHealth technology, particularly in primary care. Movida.Cronos allowed the prescription and monitoring of performance and adherence levels to the prescribed exercise program. It brings novel perspectives on prescription and remote monitoring of physical exercise, leading to an increased adherence.

